# Adolescent social media user types and their mental health and well‐being: Results from a longitudinal survey of 13–14‐year‐olds in the United Kingdom

**DOI:** 10.1002/jcv2.12071

**Published:** 2022-03-10

**Authors:** Lizzy Winstone, Becky Mars, Claire M. A. Haworth, Jon Heron, Judi Kidger

**Affiliations:** ^1^ Population Health Sciences, Medical School University of Bristol Bristol UK; ^2^ NIHR Biomedical Research Centre at the University Hospitals Bristol NHS Foundation Trust Bristol UK; ^3^ School of Psychological Science University of Bristol Bristol UK; ^4^ The Alan Turing Institute, British Library London UK

**Keywords:** anxiety, depression, latent class analysis, self‐harm, social media use, well‐being

## Abstract

**Background:**

There is mixed evidence as to the effects of different types of social media use on mental health, but previous research has been platform‐specific and has focused on an oversimplified distinction between active and passive use. This study aimed to identify different underlying subgroups of adolescent social media user based on their pattern of social media activities and test associations between user type and future mental health.

**Methods:**

Students from 19 schools (*N* = 2456) in south‐west England completed an online survey measuring 13 social media activities and four psychosocial outcomes (past year self‐harm, depression, anxiety and poor well‐being) at age 13 years (October 2019) and repeated a year later (October 2020; aged 14 years). Latent class analysis using Mplus identified distinct classes of social media user and stability of these classes was examined using latent transition analysis. A bias‐adjusted three‐step model was used to test associations between class membership at baseline and mental health at follow‐up. Analyses were adjusted for gender, ethnicity, sexual orientation, socioeconomic status, disability, social media screen‐time and baseline mental health.

**Results:**

A four‐class model of social media user at baseline was selected based on fit statistics and interpretability. User types were labelled High Communicators; Moderate Communicators; Broadcasters; and Minimal users. Users became more active over time. Broadcasters at age 13 had the poorest mental health outcomes at age 14, with mental health and well‐being generally better among the High and Moderate Communicators.

**Conclusions:**

Findings suggest that broadcasters—adolescents with high levels of content sharing in addition to messaging and browsing online—are most likely to be experiencing poor mental health a year later. Recommendations regarding social media use should expand to consider different user types, and mental health implications of their engagement with different online activities in addition to screen‐time.

## Graphical abstract

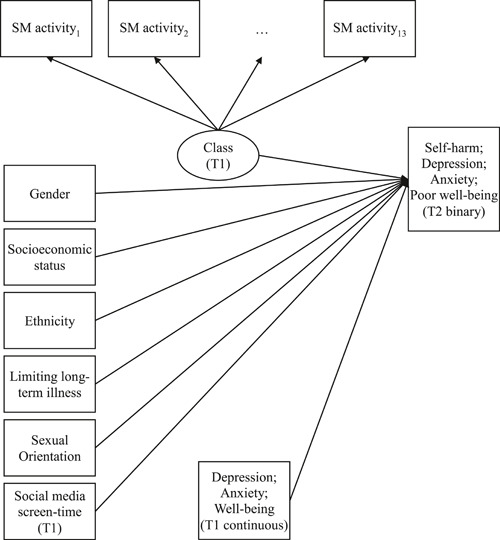

A typology of four distinct social media user groups was identified in early adolescence: High Communicators, Moderate Communicators, Broadcasters and Minimal Users. Broadcasters at age 13 ‐ young people who frequently share content online in addition to messaging and passive social media use ‐ had the poorest mental health a year later, whereas Moderate and High Communicator groups ‐ who shared content less frequently relative to other activities ‐ had the best outcomes. Digital literacy education should encourage reflection on social media activities rather than overemphasising the significance of total time spent online.

**Key points**

- Previous research on types of adolescent social media user has been platform‐specific
- Our study identifies a person‐centred, four‐class typology of non‐platform‐specific social media user in early adolescence, including High Communicators, Moderate Communicators, Broadcasters, and Minimal users
- We present evidence that Broadcasters—those who frequently share content in addition to socialising or browsing online—have the highest risk of self‐harm, anxiety, depression and poor well‐being a year later
- Clinicians should explore how and why social media is used when working with adolescents
- Digital literacy education should acknowledge the possible benefits of moderate online messaging as well as risks specific to social media broadcasting, rather than overemphasising the significance of overall time spent online

## INTRODUCTION

To date, the media effects literature has primarily focused on measuring young people's social media use in terms of frequency or screen‐time (Kaye, 2021). The prevailing narrative has tended to focus on online harms, with much of the guidance available to clinicians, parents and schools concerned with limiting screen‐time (Blum‐Ross & Livingstone, 2016). However, quantifying social media screen‐time is overly simplistic as it obscures the vast heterogeneity of social media behaviour (Kaye, 2021). As such, distinctions are now generally made between ‘active’ social media use (SMU)—direct messaging between friends, responding to content with comments or likes, and posting content to audiences of varying degrees of publicness—and ‘passive’ SMU—browsing content, silently lurking on the profiles of others or other non‐social activities. These types of use have differential relationships to mental health and well‐being, with evidence suggesting that passive SMU leads to increased depression, anxiety and poorer well‐being (Frison & Eggermont, 2020; Thorisdottir et al., 2019; Verduyn et al., 2017). According to the Passive SMU Hypothesis, upward social comparisons, leading to feelings of envy and rumination, are considered a key process in the relationship between passive SMU and poor adolescent mental health (Valkenburg et al., 2022; Verduyn et al., 2017). Increased access to social information about others can further foster feelings of social exclusion, referred to as ‘fear of missing out’ (Przybylski et al., 2013). Conversely, active SMU is thought to promote well‐being through provision of emotional support and social connectedness (Nowland et al., 2018).

Existing research has typically examined the effects of active or passive social media screen‐time on adolescent mental health. In reality, the distinction between different types of use is often blurred, with young people unlikely to engage in one type of use in isolation (Trifiro & Gerson, 2019). A person‐centred approach identifies engagement in different social media activities in different combinations, rather than grouping together similar activities in a variable‐centred approach. To date, this approach has only been used to examine time spent using specific platforms (Vannucci & Ohannessian, 2019). Given the instability in popularity and functionality of social media platforms, research with a broader focus on non‐platform‐specific SMU is important and currently lacking (Kaye, 2021). No known studies to date have adopted a person‐centred approach to identifying non‐platform‐specific types of SMU in early‐adolescence—or examined the stability of social media user types—instead using variable‐centred approaches to identify or pre‐define types of use to assess the effects on psychosocial outcomes (Frison & Eggermont, 2020; Thorisdottir et al., 2019). Prior research on social media activities has focused on a limited range of outcomes, most notably depression or well‐being. To our knowledge, no prior studies have examined associations between type of SMU and self‐harm, and few examining anxiety. These are key outcomes of interest, given increasing rates of self‐harm and emotional disorders in the UK, particularly among adolescent girls and young women (Morgan et al., 2017; Sadler et al., 2018).

## OBJECTIVES

This study will address the following research questions:

1. What are the underlying non‐platform‐specific social media user types found in a sample of adolescents?
2. Do social media user types remain stable over time (age 13–14)?
3. Do social media user types differ demographically, including gender, sexual orientation, ethnicity, socioeconomic status and limiting long term illness (LLTI)?
4. What is the relationship between social media user types at age 13 and psychosocial outcomes (self‐harm, anxiety, depression and well‐being) 1 year later?

In line with previous research in support of the Passive SMU Hypothesis, we hypothesised that the social media user types characterised by higher levels of active sharing of content and direct communication (messaging) will experience positive mental health and well‐being outcomes, and that user types characterised by higher levels of passive activities will experience more negative outcomes.

## METHODS

All non‐fee paying secondary schools across four local authorities in south‐west England, UK (*N* = 77) were invited by email to participate in the study. Of these, 30 responded and 19 schools went on to take part in the study. Recruitment stopped once 19 schools had signed up to the study based on sample size calculations designed to detect an effect size of 0.20, given previous studies on screen‐time had found only small effects. Compared to non‐participating schools in the target area, participating schools were more likely to be in urban areas, be non‐religious and have a higher percentage of students eligible for free school meals (Table S1). All Year 9 students (aged 13–14 years) were invited to complete an online survey during lesson time in October and November 2019 (available at https://osf.io/7xz6u/) and again a year later. Paper surveys were provided for schools on request. Baseline mean response rate across schools was 76.7% (SD = 15.1%), with a total sample size of 2549 at time 1 (T1) and 1459 at time 2 (T2). Data on social media activities were available for 2456 respondents at T1 (59.1% female, *M*age = 13.23 years). Of these 1311 had complete data on covariates including baseline depressive symptoms (1314 anxiety; 1321 well‐being). Despite high attrition, the included sample and potential sample were comparable across key demographics, mental health and social media screen‐time (Table S2).

### Measures

#### Social media activities

Respondents reported frequency of use of social media for 13 activities taken from the Mental Health of Children and Young People in England 2017 survey (Vizard et al., 2018): *Sending messages to people; Sharing photos or videos of yourself; Sharing photos or videos of other things; Sharing quizzes and polls; Looking at photos or videos posted by other people; Listening to music; Playing games; Finding out about things you are interested in; Expressing your views about things (e.g. by blogging or posting content); Arranging to meet friends; Creating events; Meeting new people online; Meeting new people face to face*. Answers were given on a 4‐point scale: *never, rarely, fairly often* and *very often*. Those who reported no SMU (*N* = 33 with T1 covariate data) were re‐coded as ‘never’ for each activity. Where a response category was selected by fewer than 10% of respondents, two categories were merged for example, *never* was merged with *rarely* for messaging, looking at photos and listening to music.

While the study was intentionally non‐platform‐specific, participants were briefed to think about their use of all social media platforms, including Instagram, Snapchat, WhatsApp, Facebook, Facebook Messenger, Tumblr, TikTok, Twitter, Pinterest, YouTube, Sarahah, Tellonym, House Party.

#### Self‐harm

A single item measure of self‐harm was taken from the Child & Adolescent Self‐harm in Europe Study (Madge et al., 2008): ‘Have you ever hurt yourself on purpose in any way, e.g. by taking an overdose of pills or by cutting yourself’ with the response options modified to include: *no, I have never hurt myself on purpose; yes, but not in the last year; yes, in the last year*. This was recoded for use as a binary variable to examine past‐year self‐harm.

#### Anxiety and depression

Anxiety and depression were assessed using the Hospital Anxiety and Depression Scale (HADS). The HADS consists of 14 items: seven measuring anxiety (T1 *α* = 0.85; T2 *α* = 0.87) and seven measuring depression (T1 *α* = 0.72; T2 *α* = 0.74) with reported symptoms over the past week. All items were measured using a four‐point item‐specific response scale from 0 to 3, with total scores on each sub‐scale ranging from 0 to 21. To enable inclusion in a bias‐adjusted three‐step model, both sub‐scales were recoded as binary variables. In line with recommended cut‐off points for adolescents (White et al., 1999), scores of nine or above indicate moderate risk of anxiety and seven and above moderate risk of depression.

#### Poor well‐being

The Warwick and Edinburgh Mental Well‐being Scale (WEMWBS; Tennant et al., 2007) measures general subjective well‐being over the past 2 weeks and consists of 14 positively worded items (T1 *α* = 0.91; T2 *α* = 0.92) with a five‐point response scale ranging from *none of the time* to *all of the time*. The total score (ranging from 14 to 70) was recoded as a binary variable with poor well‐being defined as one standard deviation below the T2 sample mean (44.83, SD = 11.23), that is, a WEMWBS score below 33.60.

#### Covariates

Detailed information about covariates is included in Table S3. Analyses were first unadjusted (model 0) then adjusted for key demographics (gender, socioeconomic status, ethnicity, limiting long term illness or disability (LLTI) and sexual orientation; model 1). These demographics were chosen as they are likely to be associated with mental health difficulties. In model 2 we additionally adjusted for overall estimated weekday and weekend‐day social media screen‐time at T1 as previous research has indicated that the association between SMU and poor mental health may be explained by displacement of time spent engaging in healthier activities (Viner et al., 2019). In model 3 we additionally adjusted for baseline mental health (continuous measures of depression, anxiety or well‐being).

### Analysis

**FIGURE 1:**
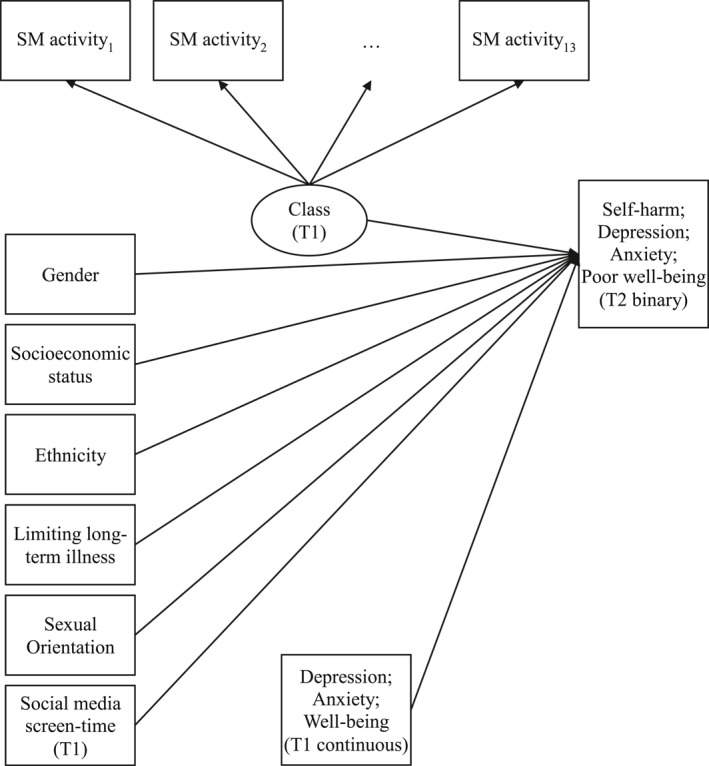
Model diagram, three‐step bias adjusted latent class analysis

Latent class analysis (LCA) was conducted using Mplus version 8.4 (Muthén & Muthén, 1998‐2015). LCA allows identification of unobserved classes (social media user types) from observed categorical multivariate data (social media activities). Class enumeration is decided through specification of model solutions with increasing number of classes, followed by comparison of fit statistics, utility and interpretability. For this study, we compared models with two to five classes, based on the 13 social media activities. Fit indices considered (Table S4) were the Bayesian information criterion (BIC), Sample‐size adjusted BIC and Akaike information criterion (AIC), with lower values indicating improved fit. The Lo‐Mendell‐Rubin adjusted likelihood ratio test (LMR LRT) compares model fit for *k* and k‐1 clusters. Model fit was considered alongside class size and classification probabilities to assess interpretability of each solution (Nylund‐Gibson & Choi, 2018). Following determination of class enumeration and adjustment for classification error, we examined proportions of demographic variables—gender, ethnicity, sexual orientation, LLTI and socioeconomic status—across classes. Class enumeration was repeated at T2 to assess stability of the class solution. Latent Transition Analysis (LTA) was conducted using the sample with class membership at both time points (*N* = 1425), examining transition probabilities to assess stability of class membership (Collins & Lanza, 2010). All models were adjusted to account for school‐based clustering. Bias‐adjusted binary logistic regression (manual implementation of a bias‐adjusted three‐step method) was used to model outcomes on latent class membership (treated as categorical indicators). T1 covariates and T2 mental health distal outcomes were incorporated (Figure 1) using the sample with complete covariate data including baseline mental health (*N* = 1311 for self‐harm and depression; 1314 for anxiety; 1321 for poor well‐being). Wald statistics were used to determine whether outcomes varied by latent class membership (Nylund‐Gibson & Choi, 2018). Coefficients were exponentiated to odds‐ratios (ORs) signifying the odds of the outcome (self‐harm, anxiety, depression and well‐being) for each latent class in six paired comparisons.

Missing data—class enumeration was conducted using the sample with at most one missing value of the social media activity items (T1 *N* = 2456; T2 *N* = 1820). Of these participants, 1311 to 1321 had complete mental health data at baseline in addition to complete demographic and screen‐time covariate data. A maximum likelihood (ML) approach was used to handle missing mental health outcome data at follow up, with estimation up to the number with complete covariate data (*N* = 85 for self‐harm; *N* = 40 for depression; *N* = 42 for anxiety; *N* = 80 for poor well‐being). This approach assumes missingness to be missing at random, conditional on the other variables in the model.

## RESULTS

### Class enumeration

A four‐class model was selected at T1 based on a combination of model fit (Table S4) and interpretability of classes (AIC = 61,328; BIC = 2019; Sample‐size Adjusted BIC = 61,640). The LMR LRT index indicated that a five‐class model did not offer improved model fit compared to a four‐class model. An entropy value of 0.81 indicated good distinction between the classes.

**FIGURE 2:**
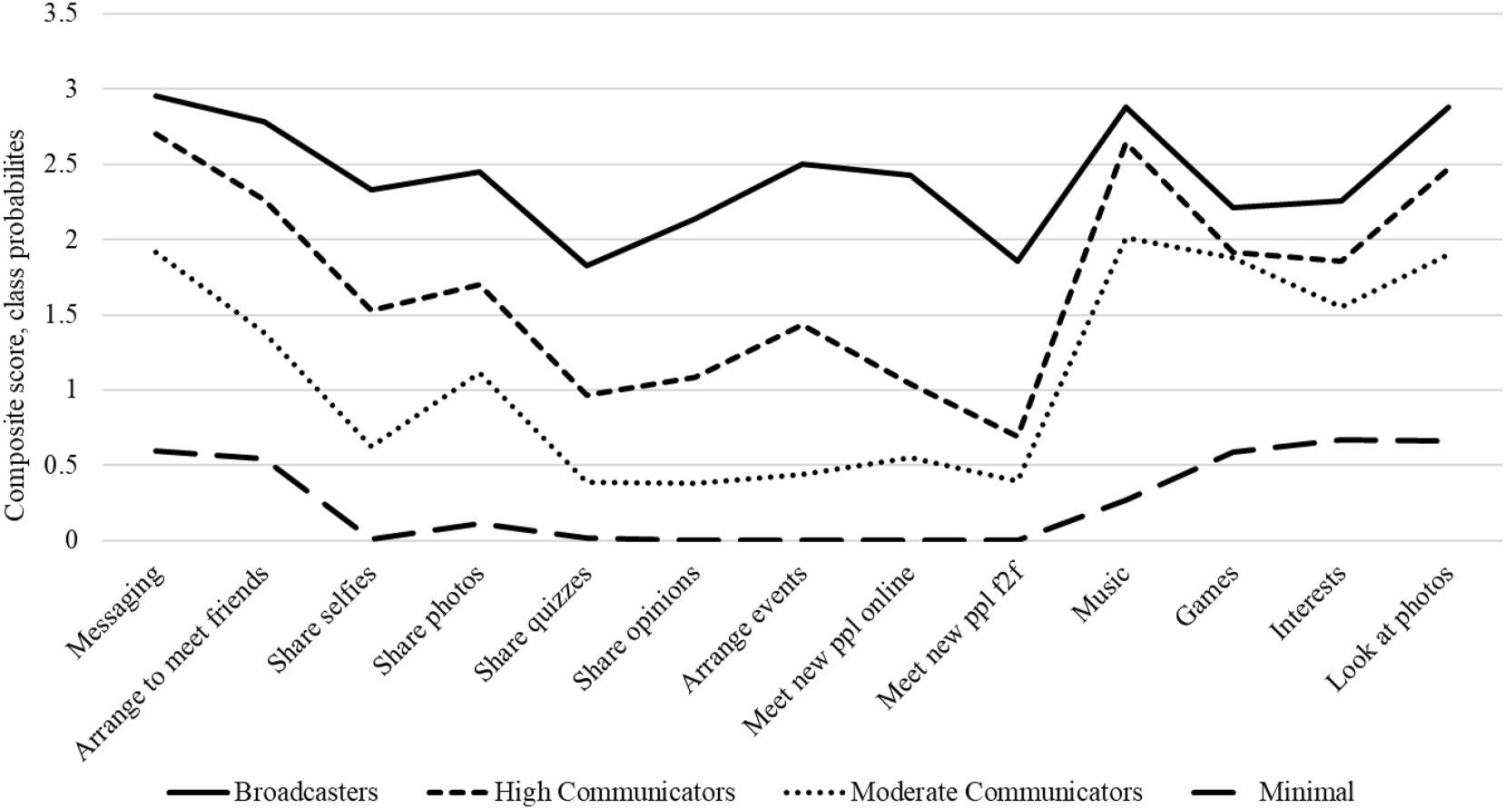
Types of adolescent social media user at T1 and composite class probability scores for different social media activities

The four classes were labelled *High Communicators* (47.8% of the sample), *Moderate Communicators* (33.1%), *Broadcasters* (12.6%) and *Minimal* (6.5%). The distinction between the non‐minimal classes relates to the intensity of messaging and differences in propensity for sharing content with others. *High Communicators* were characterised by frequent messaging and socialising with more moderate content sharing (posting selfies, opinions and other content) and browsing; *Moderate Communicators* were characterised by moderate messaging and browsing but minimal content sharing; *Broadcasters* were characterised by frequent content sharing in addition to messaging, socialising and browsing; *Minimal* users were characterised by non‐use of social media, or infrequent messaging and browsing. Composite scores based on conditional probabilities for each social media activity are displayed in Figure 2, which shows that Broadcasters were characterised by higher probabilities across all 13 SMU activities. Probabilities of messaging, arranging to meet friends, music, gaming, interests and looking at photos were only slightly higher than the High Communications class, however much larger differences were found for content sharing activities such as sharing selfies, photos, quizzes, and opinions.

### Demographic differences

Adjusting for bias, social media user type at T1 was associated with gender (Wald = 138.81, *p* < 0.001)—but not ethnicity (Wald = 2.46, *p* = 0.482), sexual orientation (Wald = 6.11, *p* = 0.106), LLTI (Wald = 0.86, *p* = 0.835) or receipt of free school meals (Wald = 4.93, *p* = 0.177).

High Communicators and Broadcasters were more likely to be female (71.4% female, (95% CI: 62.4%–80.4%) and 71.3% female (95% CI: 55.4%–87.2%) respectively), whereas Moderate Communicators and Minimal users were more likely to be male (42.3% female, (95% CI: 30.5%–54.1%) and 39.0% female (95% CI: 22.5%–55.5%) respectively).

Table S5 provides descriptive statistics for demographic covariates, screen‐time, specific platform use, and baseline mental health across T1 social media user types. Notable differences in platform use included higher use of TikTok, Facebook and Twitter in Broadcasters compared to any other user type. Moderate Communicators reported lower use of Snapchat compared to High Communicators and Broadcasters, and all three non‐minimal user types reported high use of Instagram, WhatsApp, and YouTube. Broadcasters and High Communicators reported the highest social media screen‐time and most Minimal users reported less than 30 min daily use. Baseline mental health was poorest in Broadcasters and best in Moderate Communicators and Minimal users.

### Change in social media user type, age 13–14 years

There were changes in social media user types between the two time points, with adolescents generally becoming more ‘active’ over time. The Minimal user type shrank substantially, leaving a three‐class model of users at age 14. The class profiles of the four‐class model at T1 and the three‐class model at T2 were similar. Prevalence of the High Communicators and Broadcasters classes increased over time and prevalence of the Moderate Communicators class decreased (Table 1).

**TABLE 1:** Four‐latent‐status model of social media use, T1–T2

*Assigned label*	Latent status			
	High communicators	Moderate communicators	Broadcasters	Minimal
*Probability of membership*				
T1 (age 13)	0.48	0.33	0.13	0.07
T2 (age 14)	0.54	0.11	0.35	n/a
*Probability of transitioning to…*	*…T2 latent status*			
*Conditional on T1 latent status*				
High Communicators	**0.49**	0.00	0.51	n/a
Moderate Communicators	0.78	**0.19**	0.03	n/a
Broadcasters	0.05	0.00	**0.95**	n/a
Minimal	0.27	0.70	0.03	n/a

*Note*: Diagonal transition probabilities in bold to facilitate interpretation.

### Prospective associations between latent classes of social media use and psychosocial outcomes

In fully adjusted models (model 3), social media user type at T1 was associated with all four T2 outcomes: past year self‐harm (Wald = 18.07, *p* < 0.001), anxiety (Wald = 11.45, *p* < 0.01), depression (Wald = 14.72, *p* < 0.01) and poor well‐being (Wald = 14.25, *p* < 0.01). For results, see also Table 2 and Figure S1.

**TABLE 2:** Three‐step bias‐adjusted latent class analysis, T1 social media use latent class and T2 mental health

	Model 0 OR (95% CI)	Model 1 AOR (95% CI)	Model 2 AOR (95% CI)	Model 3 AOR (95% CI)
Self‐harm, *N* = 1311				
Wald (*df* = 3)	98.52, *p* < 0.001	31.60, *p* < 0.001	41.65, *p* < 0.001	18.07, *p* < 0.001
Broadcasters versus Minimal	3.67 (1.82–7.39)	3.15 (1.57–6.31)	1.73 (0.78–3.84)	1.95 (0.92–4.14)
Broadcasters versus High Comm.	1.60 (1.05–2.42)	1.49 (0.97–2.28)	1.24 (0.78–1.99)	1.19 (0.69–2.04)
Broadcasters versus Moderate Comm.	6.21 (4.06–9.50)	5.00 (3.07–8.15)	3.51 (2.20–5.61)	3.74 (2.35–5.96)
High Comm. versus Minimal	2.30 (1.18–4.47)	2.12 (1.04–4.31)	1.39 (0.67–2.90)	1.65 (0.83–3.27)
High Comm. versus Moderate Comm.	3.89 (2.61–5.80)	3.37 (2.01–5.65)	2.83 (1.75–4.56)	3.16 (1.93–5.18)
Moderate Comm. versus Minimal	0.59 (0.27–1.30)	0.63 (0.30–1.31)	0.49 (0.22–1.11)	0.52 (0.24–1.14)
Anxiety, *N* = 1314				
Wald (*df* = 3)	44.22, *p* < 0.001	30.83, *p* > 0.001	20.68, *p* < 0.001	11.45, *p* < 0.01
Broadcasters versus Minimal	7.14 (3.73–13.66)	6.41 (3.00–13.71)	3.99 (1.91–8.35)	2.57 (1.13–5.82)
Broadcasters versus High Comm.	2.59 (1.62–4.15)	2.69 (1.67–4.31)	2.38 (1.48–3.82)	1.93 (1.04–3.57)
Broadcasters versus Moderate Comm.	4.54 (2.72–7.55)	3.40 (1.95–5.90)	2.64 (1.50–4.62)	1.39 (0.71–2.72)
High Comm. versus Minimal	2.75 (1.50–5.05)	2.39 (1.11–5.15)	1.68 (0.79–3.56)	1.33 (0.68–2.62)
High Comm. versus Moderate Comm.	1.75 (1.24–2.47)	1.26 (0.96–1.67)	1.11 (0.83–1.49)	0.72 (0.52–0.99)
Moderate Comm. versus Minimal	1.57 (0.91–2.71)	1.89 (0.92–3.86)	1.52 (0.75–3.06)	1.85 (0.97–3.54)
Depression, *N* = 1311				
Wald (*df* = 3)	52.80 *p* < 0.001	47.42, *p* < 0.001	16.42, *p* < 0.01	14.72, *p* < 0.01
Broadcasters versus Minimal	4.01 (2.45–6.57)	4.28 (2.56–7.13)	1.88 (0.87–4.09)	2.34 (1.15–4.79)
Broadcasters versus High Comm.	2.58 (1.85–3.60)	2.57 (1.79–3.67)	2.06 (1.45–2.93)	2.22 (1.45–3.39)
Broadcasters versus Moderate Comm.	2.98 (2.07–4.29)	2.71 (1.81–4.07)	1.72 (1.04–2.85)	1.83 (1.16–2.87)
High Comm. versus Minimal	1.55 (0.92–2.61)	1.67 (0.95–2.91)	0.91 (0.44–1.88)	1.06 (0.53–2.09)
High Comm. versus Moderate Comm.	1.15 (0.82–1.62)	1.06 (0.74–1.51)	0.84 (0.56–1.25)	0.82 (0.53–1.27)
Moderate Comm. versus Minimal	1.35 (0.86–2.11)	1.58 (1.01–2.45)	1.09 (0.61–1.96)	1.28 (0.78–2.11)
Poor well‐being, *N* = 1321				
Wald (*df* = 3)	19.48 *p* < 0.01	9.61, *p* < 0.05	9.34, *p* < 0.05	14.25, *p* < 0.01
Broadcasters versus Minimal	1.96 (1.10–3.49)	1.45 (0.73–2.89)	0.52 (0.54–1.46)	0.68 (0.28–1.64)
Broadcasters versus High Comm.	1.55 (0.96–2.49)	1.47 (0.90–2.41)	1.12 (0.68–1.85)	1.35 (0.78–2.36)
Broadcasters versus Moderate Comm.	4.00 (2.15–7.43)	2.97 (1.48–5.97)	1.77 (0.91–3.46)	2.42 (1.23–4.74)
High Comm. versus Minimal	1.27 (0.69–2.31)	0.99 (0.51–1.91)	0.46 (0.22–0.98)	0.50 (0.22–1.12)
High Comm. versus Moderate Comm.	2.58 (1.45–4.60)	2.02 (1.09–3.75)	1.58 (0.83–3.00)	1.78 (1.04–3.06)
Moderate Comm. versus Minimal	0.49 (0.26–0.92)	0.49 (0.24–0.99)	0.29 (0.13–0.66)	0.28 (0.13–0.60)

*Note*: Model 0: unadjusted; Model 1: +adjusted for demographic covariates; Model 2: +adjusted for screentime; Model 3: +adjusted for mental health at T1.

#### Self‐harm

Odds of self‐harm were higher for Broadcasters than for Moderate Communicators (fully adjusted Odds Ratio (AOR) = 3.74, 95% CI: 2.35–5.96), with some evidence of increased odds for Broadcasters compared to Minimal users (AOR = 1.95, 95% CI: 0.92–4.14). Odds were also higher for High Communicators compared to Moderate Communicators (AOR = 3.16, 95% CI: 1.93–5.18). Odds of self‐harm were higher for Broadcasters compared to High Communicators in unadjusted analysis (model 0) but attenuated to the null following adjustment for demographic covariates (model 1) and screen‐time (model 2). No differences were found between the Moderate Communicators and Minimal users.

#### Anxiety

Odds of anxiety were higher for Broadcasters compared to Minimal users (AOR = 2.57, 95% CI: 1.13–5.82) and High Communicators (AOR = 1.93, 95% CI: 1.04–3.57). Odds were also higher in Broadcasters compared to Moderate Communicators in model 2 (AOR = 2.64, 95% CI: 1.50–4.62) but attenuated to the null following adjustment for baseline anxiety (model 3). Odds of anxiety were higher for High Communicators than Moderate Communicators in unadjusted models (OR = 1.75, 95% CI: 1.24–2.47), but lower in fully adjusted models (AOR = 0.72, 95% CI: 0.52–0.99). There was some evidence of increased odds for Moderate Communicators compared to Minimal users (AOR = 1.85, 95% CI: 0.97–3.54). Odds of anxiety were higher for High Communicators compared to Minimal users in model 1 (AOR = 2.39, 95% CI: 1.11–5.15) but the association attenuated to the null following adjustment for screen‐time.

#### Depression

In fully adjusted models, odds of depression were higher for Broadcasters compared to any other group [Broadcasters vs. Minimal AOR = 2.34, (95% CI: 1.15–4.79); Broadcasters versus High Communicators AOR = 1.83 (95% CI: 1.16–2.87); Broadcasters versus Moderate Communicators AOR = 2.22 (95% CI: 1.45–3.39)]. There was no evidence of a difference between any of the other user types.

#### Poor well‐being

In fully adjusted models, odds of poor well‐being were higher for Broadcasters and High Communicators compared to Moderate Communicators [Broadcasters vs. Moderate Communicators AOR = 2.42 (95% CI: 1.23–4.74); High versus Moderate Communicators AOR = 1.78 (95% CI: 1.04–3.06)]. Odds were lower in Moderate Communicators than Minimal Users [AOR = 0.28 (95% CI: 0.13–0.60)].

## DISCUSSION

### Typology of social media use in adolescence

SMU encompasses a broad range of activities, but this complexity is often overlooked in the literature. We identified four distinct types of social media user at age 13, based on underlying differences in patterns of use between individuals. These were *High Communicators, Moderate Communicators*, *Broadcasters* and *Minimal* users. Almost half the young people in our sample were classified as High Communicators– typically engaging in high levels of online messaging and passive SMU, but lower levels of posting content.

There was limited alignment between these four types of social media user found here and a three‐factor structure of Facebook previously outlined, which distinguished between passive, active direct and active public SMU (e.g. Frison & Eggermont, 2020). The Broadcasters class in our study was characterised by frequent engagement with active public activities, with the two Communicators classes engaging in moderate or high levels of active direct SMU and lower levels of content sharing. Inclusion of non‐users and infrequent users of social media in our sample enabled detection of a small Minimal user type at T1, equivalent to low or no overall SMU in other studies. Contrary to our hypothesis, we did not find evidence of a class of users engaging in higher levels of passive use compared to social use. Instead, all non‐minimal types of user in our sample tended to report relatively frequent engagement with passive activities, suggesting that research testing the effect of passive SMU in isolation may be overly simplistic. The distinction between the non‐minimal classes lay instead with intensity of messaging and differences in propensity for sharing content with others. We found heterogeneity in outcomes between these user types, further justifying the need for a more nuanced consideration of SMU, beyond an active‐passive dichotomy.

### Change in social media user group, age 13–14 years

Little is known about changes in SMU patterns over time. We found an increase in broadcasting, which contradicts previous findings of decreases in broadcasting throughout adolescence and increases in messaging and passive SMU (Frison & Eggermont, 2020). These previous findings were Facebook‐specific and included 11–19‐year‐olds, encompassing different developmental stages. In our sample of younger adolescents, online broadcasting may still be a useful tool for identity exploration and maintaining relationships. Younger adolescents tend to develop and express their identity through creative online self‐presentation, with older adolescents defining their identity through the development of high‐quality online peer networks (Livingstone, 2008).

The increase in active SMU and disappearance of the Minimal user type in our sample may also be linked to the unique circumstances of the COVID‐19 pandemic. Though this may reflect an overall increase in time spent online, the Broadcasters class is distinct from the two Communicators classes in the users' willingness to share content, such as selfies and opinions. In the absence of physical socialising, online broadcasting may have represented an important avenue through which young people could seek the social and emotional affordances of peer validation and acceptance, as well as increased need for escapism (Moreno & Uhls, 2019).

### Social media user types and psychosocial outcomes

With few exceptions, across different aspects of mental health and well‐being, we found that Broadcasters experienced the poorest outcomes compared to all other user types. Comparisons between the other three user types varied according to outcome, with Moderate Communicators experiencing better well‐being and lower odds of self‐harm but higher odds of anxiety than High Communicators in fully adjusted models. For several—though not all—comparisons, associations were substantially attenuated following adjustment for screen‐time, indicating the importance of both activity patterns and time spent online in conceptualising harmful SMU. The lack of evidence for positive outcomes in Minimal users relative to Moderate Communicators is noteworthy, suggesting that moderate—but not excessive—online socialising may be constructive for peer connectedness, with subsequent benefits for well‐being (Frison & Eggermont, 2020; Kaye, 2021). This person‐centred evidence supports the ‘digital Goldilocks Hypothesis’ (Przybylski et al., 2020), whereby moderate social media screen‐time is beneficial to well‐being (compared to no use at all) but excessive use is associated with negative outcomes. Our findings suggest that optimal SMU should be additionally conceptualised as moderate messaging and passive use, with minimal broadcasting. Previous variable‐centred research has suggested that engaging in passive SMU may have negative consequences for loneliness, upward social comparison or envy (Verduyn et al., 2017). Our findings indicate that young people who most frequently engage in passive use do so in combination with frequent active SMU, and it is these adolescents (Broadcasters) who appear to be most at risk of future mental ill‐health.

Class membership varied by gender, with girls more likely than boys to belong to the High Communicators and Broadcaster user types. Previous research has indicated stronger associations between social media screen‐time and poor mental health for girls than boys (Kelly et al., 2018). Given our findings, future research might investigate whether propensity for different types of use may explain some of these gender differences.

A developmental social media affordances framework (Moreno & Uhls, 2019) encourages researchers to move away from a platform‐specific approach to focus on key attributes of social media for research findings and understanding to be transferrable as platforms evolve. The framework includes, among others, the social and identity affordances and risks offered by SMU to adolescent development. Consideration of the differential application of affordances provided by different types of SMU may elucidate the motivations and psychosocial consequences for the different user types identified here. Social affordances are offered by several aspects of SMU but may be most salient for those engaging in messaging, fostering a sense of belonging. Moreno and Uhls (2019) suggest ‘metavoicing’ as a further social affordance of broadcasting SMU, enabling users to contribute to online public social spaces and larger online communities. Although moderate broadcasting within friendship groups may be beneficial to identity development, more frequent and public broadcasting—especially among younger adolescents, who may lack skills in audience management and judging the appropriateness of content to share—exposes users to potential social risks, including negative feedback, bullying or feelings of cyber‐ostracism (Moreno & Uhls, 2019). With the media effects field preoccupied with the Passive SMU Hypothesis (Valkenburg et al., 2022), there is currently a lack of understanding of the psychosocial consequences of intensive social media broadcasting in adolescence. Our findings indicate this as an area warranting further research.

### Strengths and limitations

The current study has several strengths, including a longitudinal design, large sample, adjustment for covariates, a broad range of non‐platform‐specific social media activities and inclusion of a range of mental health outcomes. Although previous studies have shown an association between time spent on social media and self‐harm (Barthorpe et al., 2020), to our knowledge, this is the first study to quantitatively test longitudinal associations with different types of SMU.

Our focus was on early adolescence (13–14 years) as this is the minimum recommended user age of most platforms. Issues relating to identity development, peer relationships and belonging are prominent at this developmental stage, and the role of social media is thus likely to be particularly important for the psychosocial functioning of this age group (Vannucci & Ohannessian, 2019). To the best of our knowledge ours is the first study to identify social media user types at this age in terms of proclivity for different online activities. Rather than measure the effect of time spent using social media actively or passively, our study builds a picture of the types of social media user most prevalent in adolescence and how these user types vary in their subsequent mental health.

Our findings should be interpreted considering the following limitations. First, our data were collected via self‐report. Self‐reported screen‐time (a covariate in our study) tends to correlate poorly with objective measures (Ellis et al., 2019). However, this may not apply to self‐reported frequency of different social media activities—a more specific cognitive task not requiring time estimates and therefore likely subject to less ambiguity and recall bias. Second, social media activities were measured using an existing list encompassing both active and passive types of use (Vizard et al., 2018), however it is possible there may be additional activities not captured here. Defining active and passive activity can be challenging and ambiguous (Kaye, 2021). This complexity increases alongside evolving capabilities of social media platforms, with ‘looking at photos’ (typically thought of as a passive activity) an increasingly common part of messaging on Snapchat, Instagram and WhatsApp. Although our study was intentionally non‐platform‐specific, class membership is platform‐*dependent*, as users' social media activities are shaped by the functionality of the platforms used. Third, our study focused on 13–14‐year‐olds and our findings may not generalise to older adolescents. Indeed, our findings indicate that user types may change over time. Finally, despite a large overall sample, our analysis may have been underpowered to detect associations for Minimal users (6.5% of the sample).

## CONCLUSIONS

Media effects research to date has focused on social media screen‐time in adolescence, which fails to take account of the myriad activities encompassed by SMU. We provide novel evidence of a typology of SMU in early adolescence and found that (apart from Minimal users) young people tended to engage in both active and passive activities. This suggests that studies distinguishing between active and passive SMU may be overly simplistic. This study proposes an alternative to testing the effect of different types of social media screen‐time; first, deriving user types by examining patterns of simultaneous active and passive activities and second, longitudinally testing the mental health and well‐being of these user types. Our findings demonstrate the complex relationship between SMU and mental health. We found that those using social media for frequent broadcasting activities had the highest risk of self‐harm, anxiety, depression and poor well‐being one year later, identifying this group as warranting further investigation and potential intervention.

Clinicians should consider exploring how and why social media is used when working with young people who have poor mental health. Digital literacy education for young people should acknowledge the possible benefits of moderate SMU, particularly for messaging, as well as risks specific to social media broadcasting, rather than overemphasising the significance of overall time spent online. Young people should be encouraged to reflect on how they are using social media, and how this may link to their mental health.

## CONFLICTS OF INTEREST

No conflicts of interest to declare.

## AUTHOR CONTRIBUTIONS

Lizzy Winstone, Becky Mars, Claire M. A. Haworth, Jon Heron, and Judi Kidger contributed to conception and design of the study. Lizzy Winstone carried out the study, Lizzy Winstone, Jon Heron and Becky Mars analysed the data and Lizzy Winstone drafted the output. Lizzy Winstone, Becky Mars, Claire M. A. Haworth, Jon Heron, and Judi Kidger contributed to interpretation of data. Becky Mars, Claire M. A. Haworth, Jon Heron, and Judi Kidger critiqued the output for important intellectual content. All authors have read and approved the final version of the manuscript. Lizzy Winstone serves as guarantor for the contents of this paper.

## ETHICS STATEMENT

Informed consent was obtained, and the study was approved by the University of Bristol Faculty of Health Sciences Ethics Committee (Ref: 84,883).

## Supporting information

Supporting Information S1

## Data Availability

The data that support these findings are available on request from https://data.bris.ac.uk/data/dataset/9fiahitkoy8e2qxnr6a4bfuke.
