# Supplementary material for: Adolescent social media user types and their mental health and well‐being: Results from a longitudinal survey of 13–14‐year‐olds in the United Kingdom
Source: JCPP Adv. 2022 Mar 10;2(2):e12071. doi: 10.1002/jcv2.12071 (PMC10242896; doi:10.1002/jcv2.12071)
Supplement: Supplementary file 1 — Supporting Information S1 [file JCV2-2-e12071-s001.docx]

**Adolescent Social Media User Types and their Mental Health and Well-being**

**(Supporting Information)**

**Table S1. Comparison of participating and non-participating schools in the target areas (**[**https://www.compare-school-performance.service.gov.uk/**](https://www.compare-school-performance.service.gov.uk/)**; data accessed 16^th^ June 2020)**

|  | **Participating schools (n=19)** | **Non-participating schools in target area (n=57)** |
| --- | --- | --- |
| Rural | 10% | 18% |
| Urban | 90% | 82% |
| Mixed gender | 80% | 95% |
| Mean % eligible for free school meals | 11.0% | 8.7% |
| Academy status^[[1]](#footnote-1)^ | 85% | 88% |
| No religious denomination | 70% | 88% |

**Table S2. Comparison of analytic sample (N=1,311) with potential sample (N=2,456)**

|  | **Analytic sample**  **(N=1,311)** | | **Potential sample (N=2,456)** |
| --- | --- | --- | --- |
|  | **T1** | **T2** | **T1** |
| Ethnicity: % Black and Minority Ethnic | 20.3 (18.2 to 22.6) | - | 21.6 (19.9 to 23.3) |
| Limiting long-term illness: % | 14.0 (12.2 to 15.9) | - | 14.8 (13.3 to 16.3) |
| Sexual orientation: % LGBTQ+ | 19.8 (17.7 to 22.0) | - | 18.9 (17.4 to 20.6) |
| Gender: % female | 60.9 (58.2 to 63.5) | - | 57.4 (55.3 to 59.4) |
| Receipt of free school meals: % | 8.6 (7.2 to 10.3) | - | 10.1 (8.9 to 11.4) |
| Self-harm (past year) | 16.0 (14.0 to 18.2) | 19.0 (16.9-21.3) | 17.4 (15.9 to 19.0) |
| Anxiety (score ≥ 9) | 42.7 (40.0 to 45.4) | 45.9 (43.2-48.7) | 42.7 (40.7 to 44.8) |
| Depression (score ≥7) | 29.7 (27.2 to 32.2) | 31.6 (29.1-34.2) | 30.8 (28.9 to 32.7) |
| Poor well-being  (score ≤ 34.11 T1; ≤ 33.60 T2) | 14.6 (12.7 to 16.7) | 15.3 (13.4-17.5) | 15.9 (14.4 to 17.4) |
| Social media screen-time (week-day) | | | |
| <1 hour | 25.2 (23.1 to 27.5) | 14.9 (13.2-16.7) | 25.3 (23.5 to 27.8) |
| 1-3 hours | 36.8 (34.4 to 39.3) | 35.5 (33.1-38.0) | 35.6 (33.7 to 37.5) |
| 3 hours + | 38.0 (35.5 to 40.5) | 49.6 (47.0-52.2) | 39.1 (37.2 to 41.0) |
| Social media screen-time (weekend) | | | |
| <1 hour | 14.8 (13.1 to 16.7) | 9.1 (7.7-10.7) | 14.6 (12.6 to 16.7) |
| 1-3 hours | 28.5 (26.2 to 30.9) | 23.3 (21.2-25.6) | 27.6 (25.8 to 29.4) |
| 3 hours + | 56.7 (54.1 to 59.2) | 67.6 (65.1-69.9) | 57.8 (55.8 to 60.0) |

All estimates are percentages with 95% confidence intervals and do not account for school clustering.

**Table S3. Details of covariates**

| **Covariate** | **Details** |
| --- | --- |
| Gender | Respondents were asked *Which of the following best describes your gender?* Response options were: *Male, Female, Prefer to self-describe* and *prefer not to say.* Respondents who self-described or chose not to answer were excluded from the analysis. |
| Socioeconomic status | Family Affluence Scale (FAS II) is a 6-item scale including *Does your family own a car, van or truck?* And *How many times did you and your family travel out of the UK for a holiday/vacation last year?*  Each item has a tailored response scale, with total possible summed scores ranging from 0 to 13 and lower scores indicative of lower socioeconomic status.  Respondents were also asked whether they received free school meals, with those in receipt coded as 0 and those not in receipt coded 2. This was added to the FAS II to give a possible total score ranging from 0 to 15. |
| Ethnicity | Respondents were asked to select the best description of their ethnic group from the following options:  *White; Mixed/ multiple ethnic groups; Asian/ Asian British; Black/ African/ Caribbean/ Black British; Chinese; Arab; Other ethnic group*. Responses were re-coded to a binary variable White (0) and Black, Asian and Minority Ethnic (1). |
| Limiting Long Term Illness or Disability (LLTI) | Respondents were asked *Are your day-to-day activities limited because of a health problem or disability which has lasted, or is expected to last, at least 12 months?* With response options Yes, limited a lot; Yes, limited a little and No. Responses were re-coded to a binary variable no LLTI (0) and LLTI (1). Those who specified their LLTI to be *Behaviour, social or emotional difficulties* only were recoded as 0. |
| Sexual orientation | Respondents were asked to choose from the following options:  *Straight (attracted to people of the opposite gender); Gay or lesbian (attracted to people of the same gender); Bisexual or pansexual (attracted to all genders); Asexual or Aromantic (attracted to no genders); Not sure; Prefer not to say*. Responses were re-coded to a binary variable Non-LGBTQ+ (0) and LGBTQ+ (1). Those responding *Not sure* or *Prefer not to say* were included in the LGBTQ+ group following sensitivity analyses. |
| Social media screen-time | Social media screen-time was measured by two questions:  1. *When you use social media sites or apps how much time in total do you spend using them on a typical school day? Include all social media you use and remember to include time before and after school.*  2. *When you use social media sites or apps how much time in total do you spend using them on a typical weekend or holiday day?*  Response options for both weekday and week-end social media screen-time were: *Less than 30 minutes; More than 30 minutes but less than an hour; One to two hours; Two to three hours; Three to four hours; Four to five hours; Five to six hours; Six to seven hours; More than seven hours*  For the purposes of the analyses presented here, weekday and weekend social media screen-time were treated as continuous variables, with non-users added to the lowest response category (less than 30 minutes). |

**Table S4. Fit indices for time 1 class enumeration, N=2,456**

|  | **2-class model** | **3-class model** | **4-class model** | **5-class model** |
| --- | --- | --- | --- | --- |
| **Bayesian information criterion (BIC)** | 64132 | 62504 | 62019 | 61925 |
| **Sample-size adjusted BIC (aBIC)** | 63944 | 62222 | 61640 | 61452 |
| **Akaike information criterion (AIC)** | 63789 | 61988 | 61328 | 61060 |
| **Lo-Mendell-Rubin adjusted likelihood ratio test (LMR LRT)** | 4875, p<0.001 | 1853, p<0.001 | 716, p<0.01 | 327, p=0.763 |
| **Entropy** | 0.83 | 0.83 | 0.81 | 0.76 |

**Table S5. Demographic covariates, social media screen-time, platforms used and baseline mental health across user type (T1)**

|  | **Social media user type (T1)** | | | |
| --- | --- | --- | --- | --- |
|  | **High Communicators** | **Moderate Communicators** | **Broadcasters** | **Minimal** |
| Ethnicity: Black and Minority Ethnic | 20.4% | 20.5% | 23.2% | 30.2% |
| Limiting long-term illness | 15.5% | 13.7% | 12.5% | 14.0% |
| Sexual orientation: LGBTQ+ | 17.6% | 17.7% | 25.3% | 26.7% |
| Receipt of free school meals | 7.8% | 8.4% | 9.1% | 13.8% |
| Gender: female | 71.4% | 42.3% | 71.3% | 39.0% |
| Social media screen-time (week-day) | | | | |
| <30 minutes | 2.0% | 19.1% | 1.1% | 76.6% |
| 30 minutes-1 hour | 10.5% | 20.2% | 1.8% | 8.8% |
| 1-2 hours | 20.4% | 26.6% | 6.9% | 3.8% |
| 2-3 hours | 22.3% | 12.0% | 14.3% | 5.5% |
| 3-4 hours | 16.6% | 8.4% | 21.6% | 0.9% |
| 4-5 hours | 12.6% | 6.1% | 16.2% | 2.2% |
| 5-6 hours | 11.1% | 3.3% | 9.5% | 0.0% |
| 6-7 hours | 2.8% | 1.7% | 15.0% | 1.1% |
| >7 hours | 1.6% | 2.7% | 13.7% | 1.1% |
| Social media screen-time (weekend) | | | | |
| <30 minutes | 0.0% | 9.5% | 2.3% | 73.5% |
| 30 minutes-1 hour | 2.9% | 13.9% | 1.0% | 5.5% |
| 1-2 hours | 11.0% | 20.7% | 4.0% | 7.6% |
| 2-3 hours | 16.2% | 17.5% | 6.9% | 4.1% |
| 3-4 hours | 16.7% | 10.7% | 13.1% | 0.8% |
| 4-5 hours | 13.5% | 8.1% | 12.1% | 3.3% |
| 5-6 hours | 14.7% | 6.1% | 9.6% | 1.0% |
| 6-7 hours | 10.7% | 4.5% | 11.9% | 3.4% |
| >7 hours | 14.4% | 8.9% | 39.2% | 0.9% |
| Platforms used (ever) | | | | |
| Snapchat | 80.0% | 48.3% | 90.7% | 7.1% |
| Instagram | 96.2% | 76.9% | 96.1% | 12.6% |
| TikTok | 64.9% | 30.8% | 81.1% | 5.6% |
| Facebook | 32.0% | 20.0% | 49.1% | 5.3% |
| WhatsApp | 80.0% | 78.4% | 81.7% | 18.4% |
| YouTube | 99.1% | 96.6% | 99.0% | 31.1% |
| Twitter | 24.7% | 15.3% | 40.4% | 4.1% |
| Baseline (T1) mental health |  |  |  |  |
| Self-harm in past year | 19.5% | 8.4% | 33.9% | 3.4% |
| Anxiety score ≥ 9 | 50.8% | 27.1% | 67.9% | 24.6% |
| Depression score ≥ 7 | 30.8% | 26.0% | 43.0% | 22.1% |
| Well-being score ≤ 1 standard deviation below sample mean | 15.6% | 14.3% | 18.3% | 8.1% |

All estimates are bias-adjusted and account for school clustering.


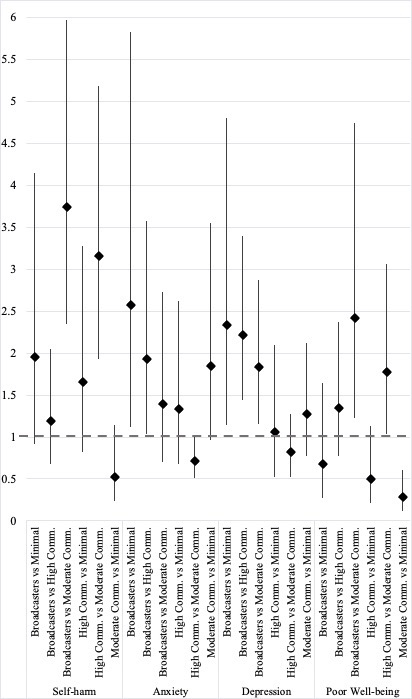


**Figure S1. Fully adjusted odds ratios (vertical lines represent 95% confidence intervals) for paired associations with four mental health outcomes at time 2**

1. Academies are state-funded schools run by not-for-profit trusts. They are not required to follow the national curriculum but are subject to the same admissions and examinations processes as other state funded schools. [↑](#footnote-ref-1)
